# Unfractionated heparin versus nafamostat mesylate for anticoagulation during continuous kidney replacement therapy: an observational study

**DOI:** 10.1186/s12882-023-03060-1

**Published:** 2023-01-16

**Authors:** Shinya Kameda, Tomoko Fujii, Junpei Ikeda, Akira Kageyama, Toshishige Takagi, Naoki Miyayama, Kengo Asano, Arata Endo, Shoichi Uezono

**Affiliations:** 1 Intensive Care Unit, The Jikei University Hospital, 3-19-18 Nishi-Shimbashi, Minato-Ku, 105-8471 Tokyo, Japan; 2 Department of Clinical Engineering Technology, The Jikei University Hospital, Tokyo, Japan; 3 Department of Pharmacy, The Jikei University Hospital, Tokyo, Japan; 4 Department of Anesthesiology, The Jikei University Hospital, Tokyo, Japan

**Keywords:** Acute kidney injury, Continuous kidney replacement therapy, Nafamostat mesylate, Unfractionated heparin sodium, Filter life, Anticoagulant

## Abstract

**Background:**

Unfractionated heparin sodium and nafamostat mesylate have long been used as anticoagulants in continuous kidney replacement therapy (CKRT) where citrate is unavailable. This study aimed to determine whether heparin or nafamostat mesylate used during CKRT was associated with a longer filter life.

**Methods:**

In this single-centre observational study, we included adult patients who required CKRT and used heparin or nafamostat mesylate for their first CKRT in the intensive care unit from September 1, 2013, to December 31, 2020. The primary outcome was filter life (from the start to the end of using the first filter). We used propensity score matching to adjust for the imbalance in patients’ characteristics and laboratory data at the start of CKRT and compared the outcomes between the two groups. We also performed restricted mean survival time analysis to compare the filter survival times.

**Results:**

We included 286 patients, 157 patients on heparin and 129 patients on nafamostat mesylate. After propensity score matching, the mean filter life with heparin was 1.58 days (*N* = 91, Standard deviation [SD], 1.52) and with nafamostat mesylate was 1.06 days (*N* = 91, SD, 0.94, *p* = 0.006). Multivariable regression analysis adjusted for confounding factors supported that heparin was associated with a longer filter life compared with nafamostat mesylate (regression coefficient, days, 0.52 [95% CI, 0.15, 0.89]). The between group difference of the restricted mean filter survival time in the matched cohort was 0.29 (95% CI, 0.07–0.50, *p* = 0.008).

**Conclusion:**

Compared to nafamostat mesylate, heparin was associated with one-third to one-half a day longer filter life.

**Trial registration:**

Not applicable.

**Supplementary Information:**

The online version contains supplementary material available at 10.1186/s12882-023-03060-1.

## Introduction

Acute kidney injury (AKI) is frequently observed in intensive care units (ICU) and is associated with a high mortality rate. Recent epidemiological studies have shown that approximately 40 – 50% of ICU patients have AKI and that mortality increases with the severity of AKI [1–3].

Continuous kidney replacement therapy (CKRT) is usually prescribed, assuming the filter can be used continuously for at least 24 h. However, treatment is often interrupted to change the filter due to clotting in the dialysis circuit. Frequent circuit clotting increases the patient's blood loss and the staff’s workload, and prolonged interruptions reduce the therapeutic efficacy of CKRT [4].

The major anticoagulants used globally to prevent clotting in CKRT are heparin sodium and sodium citrate [5, 6]. Other anticoagulants used during CKRT include nafamostat mesylate (NM). NM, a synthetic serine proteinase inhibitor (molecular weight 539.6), is often used, especially in Japan and Korea [7, 8], to provide anticoagulant effects by blocking the blood coagulation cascade inhibiting complement-mediated haemolysis. It has a short half-life of approximately 8 min; thus, it is supposed to act regionally in the circuit [9, 10]. Since COVID-19 became a pandemic, in which thromboembolic complications are common and some patients need extracorporeal membrane oxygenation, the property of NM as an anticoagulant has attracted clinicians in various countries [11–13].

Despite the favourable features of NM for use in CKRT, there has been little research to assess its clinical utility [14]. A recent systematic review reported that when comparing the NM and no-anticoagulant groups, some studies found more major bleeding in the NM group than in the no-anticoagulant group, no significant difference in the 28-day mortality between the two groups, and no difference in renal function recovery or catheter thrombotic events. However, there are insufficient studies to form an evidence base for comparing NM and heparin [15]. Due to the paucity of clinical evidence on its effectiveness and safety, the international guideline does not mention NM as an anticoagulant for CKRT [14].

This study aimed to explore the comparative effectiveness of unfractionated heparin (UFH) and NM on the filter life of CKRT in ICU patients.

## Methods

### Study design, setting, and participants

We conducted a single-centre, retrospective, observational study at a 20-bed general ICU. A STROBE checklist was used to report the study results.

We screened all critically ill adult patients who required CKRT during their ICU stays from September 2013 to December 2020. We included patients who received UFH or NM during CKRT. Patients who opted out of participating were excluded. Data of the first filter used in the first CKRT for each patient were sent to the analysis.

### Data sources and variables

We collected the following data from electronic medical records and a local ICU database: demographic information (age, sex, height, weight), past medical history (hypertension, maintenance dialysis, ischemic heart disease, heart failure in NYHA-4 (New York Heart Association-4), diabetes requiring insulin, respiratory failure, and liver cirrhosis), the reason for ICU admission, emergency or scheduled admission, acute physiology and chronic health evaluation (APACHE) II score, medical or surgical admission, nephrotoxic agents use, and bleeding risks.

Bleeding risk was defined, based on definitions used in previous studies [15, 16], as any of the following conditions at CKRT initiation; within 48 h of a haemorrhagic event, within 48 h of surgery, within 4 weeks of cerebral haemorrhage, within 2 weeks of stroke, activated partial thromboplastin time (aPTT) > 60 s, prothrombin time-international normalised ratio (PT-INR) > 2.0, and platelet count < 100,000/μL. CKRT information was collected, including blood flow rate, dialysate flow rate, and body fluid removal rate. Laboratory data including arterial blood gas analysis, haemoglobin, platelet count, PT-INR, aPTT, bilirubin, creatinine, blood urea nitrogen, estimated glomerular filtration rate, C-reactive protein (CRP), and white blood cell (WBC) count at CKRT start time and 24 h later were collected.

The primary outcome was filter life, defined as the time from the CKRT initiation to the end of the first filter use due to filter clotting. If the filter was terminated for reasons unrelated to anticoagulation (e.g., leaving the ICU for imaging examination, surgical operation, or discharge), it was treated as censored. The secondary outcomes were ICU length of stay, hospital length of stay, duration of mechanical ventilation, dialysis dependence at hospital discharge, creatinine at hospital discharge in non-dialysis dependent patients, the amount of blood transfusion within the first 48 h of CKRT, ICU mortality, hospital mortality, and inflammatory biomarkers (WBC and CRP).

### The standard prescription of CKRT at the study site

The CKRT mode was continuous haemodialysis, and polysulfone or cellulose triacetate membranes were used by default at the study site. The anticoagulant was administered by continuous infusion at 400 U/h for UFH or 10 mg/h for NM.

### Statistical methods

Continuous data were presented as means with standard deviations, and count data were presented as absolute numbers with percentages. The association between the anticoagulant choice and outcomes was estimated using propensity score (PS)-matched analyses. The PS for each patient to receive NM was calculated using a logistic regression model, with the dependent variable being NM administration and the following independent variables: age, sex, chronic maintenance haemodialysis, APACHE II score, bleeding risk, laboratory data at the start of CKRT (haemoglobin, platelet, aPTT, PT-INR, WBC, and CRP), and admission for cardiovascular diseases. The selection of the variables was based on clinical relevance, imbalances observed in the baseline data and the commonly used variables in the clinical research in critical care field. NM has an anti-inflammatory property as a protease inhibitor and is also approved for acute pancreatitis [17]. WBC and CRP were, therefore, assessed in this study. The distribution of covariates was compared between the two anticoagulant groups in a 1:1 matched sample using standardised differences. Variables with standardised differences < 0.1 were determined to be well-balanced. The association between anticoagulants and outcomes was assessed using generalised linear regression after performing PS matching. In the model, the dependent variable was each outcome, and the independent variable was the anticoagulant. The results were reported as risk ratios or mean differences with their 95% confidence intervals (CI).

To account for censoring, i.e., termination of filter for reasons other than filter clotting, we used Cox proportional hazards models to assess the association between anticoagulant choice and filter life. The results were presented as hazard ratios and 95% CI along with Kaplan–Meier curves.

If PS matching did not achieve a well-balanced distribution between the two groups, a generalised linear regression analysis incorporating the imbalanced factor(s) as the independent variable(s) was added. The results were reported as adjusted risk ratios or mean differences with their 95% CI.

Furthermore, we conducted restricted mean survival time analysis, taking into account that the observation of the filter life was frequently censored after 2 days of CKRT likely due to renal recovery or other reasons, which could violate the proportionality of the hazards [18, 19]. The restricted mean filter survival times were reported with their 95% CI and the between group difference was also reported.

### Sensitivity analysis

We performed a standardised mortality ratio weighted analysis of the generalised linear model to determine the robustness of the findings we obtained from the PS matching analysis [20]. We calculated the standardised mortality ratio weights (SMRW) as 1 for NM administration and as PS/(1-PS) for the other [21]. The risk model of the choice of anticoagulants was developed in the weighted population.

We additionally performed post-hoc sensitivity analyses in the matched cohort. We calculated the inverse probability of censoring weights to account for the possible informative censoring, and the primary outcome was assessed using the generalised linear model incorporating the weights. In addition, post-hoc power calculations were performed as the study did not calculate sample size and used all available data to maximise the information size, given the exploratory nature of the study.

All analyses were performed using R ver.4.1.1 (R Foundation for Statistical Computing, Vienna, Austria). *P*-value < 0.05 was considered to be statistically significant.

## Results

### Patient characteristics and CKRT data

During the study period, 597 critically ill patients required CKRT. We excluded 311 patients who received other anticoagulants or no anticoagulants; a total of 286 patients were included in this study. One hundred and twenty-nine patients received NM, and 157 patients received UFH.

The mean patient age was 68 years in both groups (standard deviation [SD] 12 in the NM group; 13 in the UFH group), and the APACHE II score was not materially different between the two groups. Patients in the UFH group were more likely to undergo elective surgeries, particularly cardiovascular surgery. More patients had ischemic heart disease and chronic maintenance dialysis in the UFH group (Table 1).

**Table 1:** Baseline characteristics of the patients

	Nafamostat mesylate (*n* = 129)	Unfractionated heparin (*n* = 157)	*p*-value
Age, years	68 (12)	68 (13)	0.737
Sex			0.271
Male, n (%)	92 (71)	122 (78)	
Female, n (%)	37 (29)	35 (22)	
Height, cm	162.0 (10.2)	163.6 (8.9)	0.176
Weight, kg	59.6 (19.1)	60.5 (13.2)	0.639
Admission type, n (%)			< 0.001
Medical	85 (65.9)	69 (43.9)	
Emergency surgery	26 (20.2)	24 (15.3)	
Elective surgery	18 (14.0)	64 (40.8)	
Cardiovascular surgery, n (%)	47 (36.4)	98 (62.4)	< 0.001
Admission route, n (%)			< 0.001
Ward	63 (48.8)	38 (24.2)	
Operation room	39 (30.2)	81 (51.6)	
Emergency room	26 (20.2)	34 (21.7)	
Other hospital	1 (0.8)	4 (2.5)	
APACHE II score	28 (7)	27 (7)	0.061
Chronic medical condition			
Hypertension, n (%)	99 (76.7)	134 (85.4)	0.087
Ischemic heart disease, n (%)	43 (33.3)	80 (51.0)	0.004
Diabetes mellitus, n (%)	18 (14.0)	19 (12.1)	0.774
Maintenance dialysis, n (%)	56 (43.4)	102 (65.0)	< 0.001
Heart failure, n (%)	3 (2.3)	2 (1.3)	0.824
Respiratory failure, n (%)	2 (1.6)	3 (1.9)	1.000
Liver failure, n (%)	2 (1.6)	0 (0.0)	0.394
Liver cirrhosis, n (%)	9 (7.0)	2 (1.3)	0.029
Bleeding risk, n (%)	56 (43.4)	82 (52.2)	0.172

Values are presented as means with standard deviations otherwise specified

APACHE II denotes acute physiology and chronic health evaluation II

**Fig. 1:**
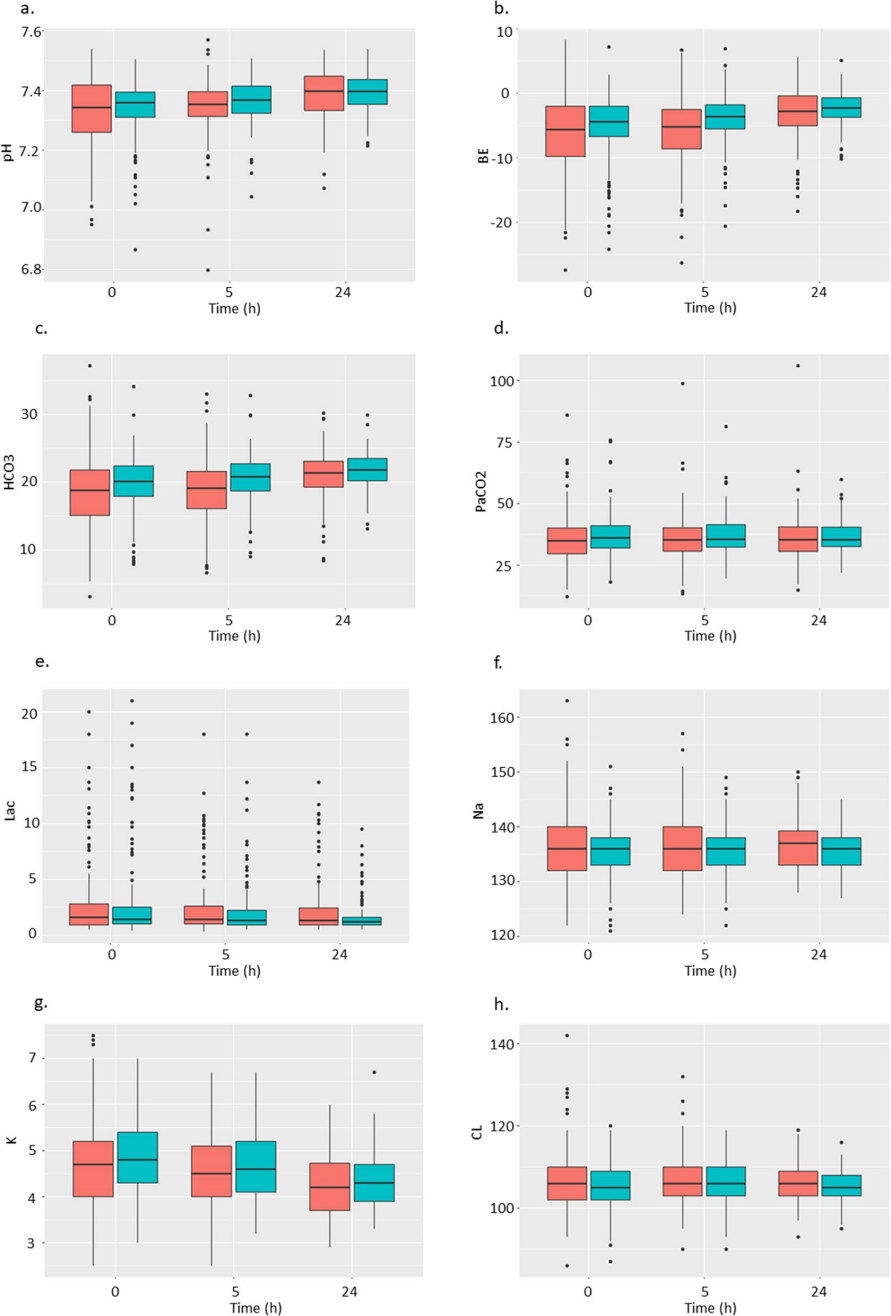
The trend of acid–base profile. Red boxes, the NM group; green boxes, the UFH group. **a**. pH, **b**. base excess, **c**. bicarbonate, **d**. PaCO2, **e**. lactate, **f**. sodium, **g**. potassium, **h**. chloride

Laboratory data at the start of CKRT were similar between the two groups (Table 2). However, vital signs and acid–base profile showed that patients in the UFH group manifested as more acidotic, with a higher heart rate than those in the NM group; thus, they received CKRT with a higher dialysate flow (Supplemental appendix, eTable 1). Protamine was not used in any of the patients in the UFH group. The trend of the acid–base profile over the first 24 h is presented in Fig. 1 (Supplemental appendix, eTable 2).

**Table 2:** Laboratory data at the start of CKRT and at 24 h

	Baseline			24 h		
	Nafamostat mesylate (*n* = 129)	Unfractionated heparin (*n* = 157)	*p*-value	Nafamostat mesylate (*n* = 129)	Unfractionated heparin (*n* = 157)	*p*-value
Total bilirubin, mg/dL	2.2 (4.5)	1.7 (4.4)	0.432	2.4 (4.6)	2.0 (4.9)	0.499
Albumin, g/dl	2.5 (0.6)	2.6 (0.6)	0.233	2.3 (0.5)	2.4 (0.5)	0.032
Uric acid, mg/dL	6.1 (3.1)	6.00(2.8)	0.724	4.7 (2.2)	4.6 (2.1)	0.528
Blood urea nitrogen, mg/dL	58 (34)	56 (28)	0.458	53 (30)	49 (21)	0.230
Serum creatinine, mg/dL	4.92 (3.48)	5.60 (3.44)	0.102	3.97 (2.50)	4.59 (2.57)	0.041
Estimated GFR, ml/min/1.73m^2^	18.1 (18.9)	17.1 (21.8)	0.688	19.2 (17.1)	18.1 (22.6)	0.648
Ca, mg/dL	7.9 (1.2)	8.1 (0.9)	0.146	8.2 (0.8)	8.3 (0.8)	0.304
Mg, mg/dL	2.3 (0.7)	2.3 (0.6)	0.716	2.3 (0.5)	2.2 (0.4)	0.345
IP, mg/dL	5.2 (2.2)	4.9 (2.0)	0.208	4.5 (1.6)	4.2 (1.6)	0.155
Hemoglobin, g/dL	9.7 (2.2)	10.1 (1.9)	0.109	9.1 (1.8)	9.3 (1.6)	0.253
Platelet, × 10^3^/μl	153.3 (98.6)	153.3 (79.6)	0.996	120.0 (83.2)	128.3 (76.5)	0.383
PT-INR > 1.5, n, (%)	12 (9.3)	18 (11.5)	0.689	12 (9.3)	17 (10.8)	0.819
APTT, sec			0.300			0.088
< 40, n (%)	92 (71.3)	101 (64.3)		58 (45.0)	61 (38.9)	
40 – 80, n (%)	36 (27.9)	52 (33.1)		60 (46.5)	90 (57.3)	
> 80, n (%)	1 (0.8)	4 (2.5)		11 (8.5)	6 (3.8)	

Values are presented as means with standard deviations, otherwise specified

*GFR* glomerular filtration rate, *PT-INR* prothrombin time-international normalised ratio, *APTT* activated partial thromboplastin time

### Outcomes

In the overall study population, the mean filter life was 1.57 days (SD, 1.51) in the UFH group and 0.97 days (SD, 0.89) in the NM group. In addition, terminations for reasons other than filter clotting were more common in the UFH group (Table 3).

**Table 3:** Secondary outcomes before and after propensity score matching

	Before propensity score matching		After propensity score matching		
	Nafamostat mesylate (*n* = 129)	Unfractionated heparin (*n* = 157)	Nafamostat mesylate (*n* = 91)	Unfractionated heparin (*n* = 91)	*p*-value
ICU mortality, n (%)	33 (25.6)	21 (13.4)	24 (26.4)	14 (15.4)	0.101
Hospital mortality, n (%)	52 (40.3)	42 (26.8)	36 (39.6)	29 (31.9)	0.353
ICU length of stay, day	11.0 (15.3)	8.4 (12.2)	12.4 (17.3)	9.5 (13.0)	0.193
Hospital length of stay, day	73.8 (69.9)	75.5 (89.8)	78.8 (74.8)	77.4 (71.8)	0.895
Mechanical ventilation days, day	5.7 (12.6)	4.1 (11.3)	6.5 (14.3)	4.6 (11.9)	0.342
Transfusion within 48 h of CKRT					
Red blood cells (ml)	122 (242)	92 (230)	112(238)	112 (238)	0.729
Platelet concentrate(ml)	26 (95)	20 (69)	30 (104)	18 (68)	0.352
Fresh frozen plasma (ml)	82 (371)	18 (120)	108 (432)	24 (120)	0.072
C-reactive protein at 24 h, mg/dL	12 (10)	13 (10)	12.71 (10.51)	12.96 (10.21)	0.873
White blood cell at 24 h, × 10^2^/μL					0.155
< 40, n (%)	5 (3.9)	2 (1.3)	5 (5.5)	2 (2.2)	
40 – 90, n (%)	34 (26.4)	66 (42.0)	27 (29.7)	38 (41.8)	
90 < , n (%)	90 (69.8)	89 (56.7)	59 (64.8)	51 (56.0)	
Dialysis dependence at discharge^a^, n (%)	50/77 (64.9)	99/115 (86.1)	40/55 (72.7)	51/62 (82.3)	0.310
Creatinine at discharge^b^, mg/dL	1.70 (1.08)	1.63 (1.04)	2.06 (1.08)	1.31 (0.74)	0.061

Values are presented as means with standard deviations, otherwise specified

*ICU* intensive care unit, *CKRT* continuous kidney replacement therapy

^a^The outcome was assessed in patients who survived to hospital discharge

^b^The outcome was assessed in patients who survived to hospital discharge without dialysis dependence

Among the secondary outcomes, ICU and in-hospital mortality were significantly higher in the NM group. Serum creatinine level at hospital discharge among patients who survived without dialysis dependence was higher in the NM group compared to the UFH group. Finally, more fresh-frozen plasma was transfused in the NM group than in the UFH group.

### PS matched cohort

**Fig. 2:**
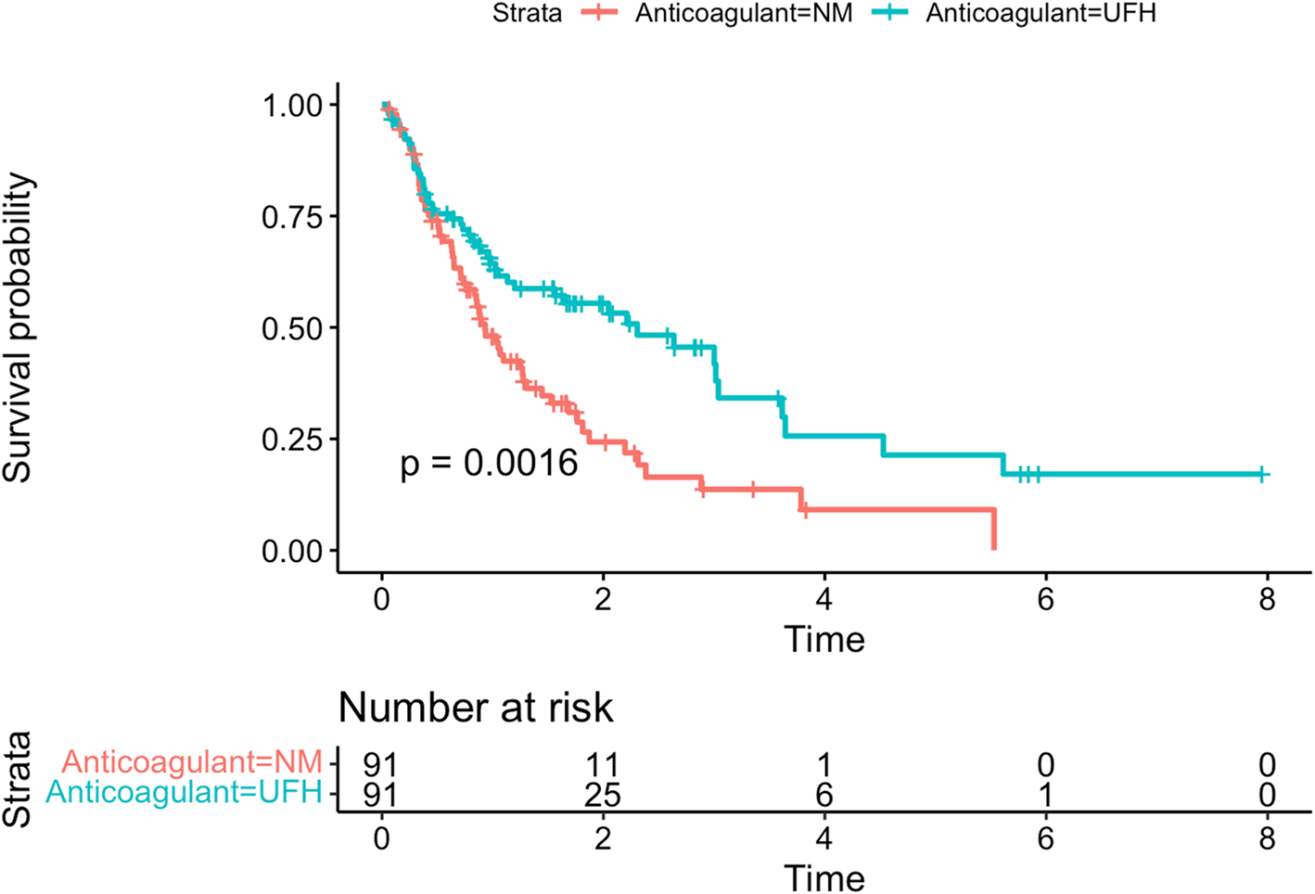
The probability of filter survival by the anticoagulant group plotted in the Kaplan–Meier curve

PS matching retrieved 91 patients from each anticoagulant group. Standardised differences indicated that the covariates were well-balanced except for aPTT at baseline (Supplemental appendix, eFigure 1). Filter life in the matched cohort was longer in the UFH group than in the NM group (1.06 [SD, 0.94] vs. 1.58 [SD, 1.52], *p* = 0.006). The additional analysis adjusting for the baseline aPTT imbalance using the generalised linear model did not change the findings (mean difference, 0.51 days, 95% CI: 0.14–0.88, *p* = 0.007). Figure 2 shows the probability of filter survival by the anticoagulant group plotted in the Kaplan–Meier curve. The hazard ratio calculated using the Cox-proportional hazard model was 0.54 (95% CI: 0.37–0.80, *p* = 0.0016).

The restricted mean filter survival time in the matched cohort was longer in the UFH group than in the NM group (1.09 [SD, 0.14] vs. 1.38 [SD, 0.15]) and the between group difference was 0.29 (95% CI, 0.07–0.50, *p* = 0.008).

The secondary outcomes were not proven to be different between the two groups (Table 3).

### Sensitivity analysis

The sensitivity analysis using an SMRW analysis supported that the UFH group had a longer filter life than the NM group (mean difference, 0.53 days [95% CI: 0.09–0.97]). The results for the secondary outcomes were also similar to the primary analysis (Supplemental appendix, eTable 3). Post-hoc sensitivity analyses also supported the findings of the primary analysis (Supplemental appendix, eTable 4).

## Discussion

### Summary of key findings

NM was more likely to be administered when patients were admitted to the ICU for medical reasons and were not on maintenance haemodialysis. Post-cardiovascular surgery patients were more likely to receive UFH than NM during CKRT. The filter life was shorter in the NM group than in the UFH group by one third to one half of a day.

### Context with prior literature

Previous studies have compared the effects of no-anticoagulant and NM on filter life during CKRT [22, 23]. Studies have also compared the effects of citrate anticoagulation and NM on filter life and bleeding during CKRT in children [24] and the effects of NM and UFH on coagulation and bleeding events during veno-arterial extracorporeal membrane oxygenation [12, 25, 26]. However, few studies have directly compared the effects of NM and UFH on filter life in CKRT. A systematic review reported no randomised clinical trials that compared the effect of UFH and NM for CKRT [15]. Two small observational studies suggested that NM might have a lower incidence of bleeding complications and a comparable or longer filter life than UFH. However, both studies were very small (101 and 81 patients) and had a high risk of errors and biases [27, 28]. The current study assessed the largest population to date to compare UFH and NM during CKRT. Another study compared NM and heparin during CKRT in patients with cerebral haemorrhage; however, the study population differed from this study [29].

A previous study reported that the filter life of NM ranged from 22 to 25.5 h [30]. The filter life of NM in this study was similar to previous studies; however, the previous research reported longer filter life for NM than UFH. The possible explanations for this discrepancy may include differences in underlying patient conditions, prescription of CKRT, and the dose of anticoagulants.

Patients with cerebral haemorrhage in the previous study [29] were less critically ill. In contrast, the current study population included ICU patients, comprising postoperative and more severely ill patients, and the assessment of bleeding risks. The fluid removal rate was lower in the NM group in the previous study [28], while there was no difference in the current study. Most importantly, the difference in patients' conditions and prescriptions was not adjusted in the analysis; thus, the baseline imbalance could have strongly biased the results. We adjusted for such confounders using PS matching analysis and confirmed the robustness of the results with an additional sensitivity analysis.

The standard prescription of UFH was 400 U/h, and that of NM was 10 mg/h at the study site. However, the optimal dose of NM is yet to be determined and should be investigated in further studies.

### Limitations

There are several limitations to this study. First, this was a single-centre study, limiting our findings’ generalizability. Due to local regulations, a relatively low dose of dialysate is prescribed for CKRT in Japan. Second, due to the study's retrospective nature over a long period, details of the delivered doses of anticoagulants were unavailable. As there is no consensus or evidence for the optimal amount of NM during CKRT, the delivered dose of NM at the study site might have been low to take good effect in the CKRT circuit. The manufacturer’s brochure includes a recommended doses of 20–50 mg/hr for haemodialysis; however, no data are available to support the doses, particularly in critical care settings. Clinicians may use the drug cautiously due to the scarcity of clinical data. Indeed, NM was used with less doses than the recommended dose in most previous studies (0.1 mg/kg/h to 0.25 mg/kg/h [28, 31], 10 mg/h [22], 20 mg/h [9, 23]). The optimal dose of NM during CKRT in the ICU needs further investigation. In turn, the amount of UFH might have been increased for other indications, such as therapeutic anticoagulation for atrial fibrillation, prosthetic valves, or deep vein thrombus. Finally, this observational study's findings should be considered exploratory, as there might be bias due to unmeasured confounders or unmet assumptions in the models. These findings should encourage future randomised clinical trials investigating the effects of NM and UFH on the design and key parameters.

## Conclusions

In this observational study, UFH was associated with one-third to one-half a day longer filter life than NM. The optimal dose of NM should be investigated before randomised clinical trials are conducted to assess its effect on CKRT.

## Supplementary Information

**Additional file 1: eFigure 1. **Standardized mean differences of variables before and after propensity score matching.** eFigure 2.** Distribution of propensity scores. **eTable 1.** Dialysis settings and vital signs at the start of CKRT. **eTable 2.** Arterial blood gas analysis at the start of CKRT, 5 hours, and 24 hours (mean SD). **eTable 3.** Sensitivity analysis with SMRW data (reference group = nafamostat mesylate). **eTable 4.** Post-hoc sensitivity analyses and power calculations.

## Data Availability

The datasets used and analysed during the current study are available from the corresponding author upon reasonable request.

## Abbreviations

AKI: Acute kidney injury
ICU: Intensive care unit
CKRT: Continuous kidney replacement therapy
NM: Nafamostat mesylate
UFH: Unfractionated heparin sodium
WBC: White blood cell
CRP: C-reaction protein
CI: Confidence interval
NYHA: New York Heart Association
APACHE: Acute physiology and chronic health evaluation
aPTT: Activated partial thromboplastin time
PT-INR: Prothrombin time-international normalised ratio
PS: Propensity score
SMRW: Standardised mortality ratio weights

## References

[1] Hoste EA (2015). Epidemiology of acute kidney injury in critically ill patients: the multinational AKI-EPI study. Intensive Care Med.

[2] Nisula S (2013). Incidence, risk factors and 90-day mortality of patients with acute kidney injury in finnish intensive care units: the FINNAKI study. Intensive Care Med.

[3] Fujii T (2018). Diagnosis, management, and prognosis of patients with acute kidney injury in japanese intensive care units: the JAKID study. J Crit Care.

[4] Uchino S (2003). Continuous is not continuous: the incidence and impact of circuit “down-time” on uraemic control during continuous veno-venous haemofiltration. Intensive Care Med.

[5] Uchino S (2007). Continuous renal replacement therapy: a worldwide practice survey. The beginning and ending supportive therapy for the kidney (B.E.S.T. kidney) investigators. Intensive Care Med.

[6] Tolwani AJ, Wille KM (2009). Anticoagulation for continuous renal replacement therapy. Semin Dial.

[7] Shinoda T (2010). Anticoagulation in acute blood purification for acute renal failure in critical care. Contrib Nephrol.

[8] Hanafusa N (2015). Application of continuous renal replacement therapy: what should we consider based on existing evidence?. Blood Purif.

[9] Choi JY (2015). Nafamostat Mesilate as an anticoagulant during continuous renal replacement therapy in patients with high bleeding risk: a randomized clinical trial. Med (Baltim).

[10] Nakae H, Tajimi K (2003). Pharmacokinetics of nafamostat mesilate during continuous hemodiafiltration with a polyacrylonitrile membrane. Ther Apher Dial.

[11] Ji HL (2021). Fibrinolytic or anti-plasmin (nafamostat) therapy for COVID-19: a timing challenge for clinicians. Pulm Pharmacol Ther.

[12] Sanfilippo F (2022). Use of nafamostat mesilate for anticoagulation during extracorporeal membrane oxygenation: a systematic review. Artif Organs..

[13] Osawa I (2021). Dynamic changes in fibrinogen and D-dimer levels in COVID-19 patients on nafamostat mesylate. J Thromb Thrombolysis.

[14] Kidney Disease: Improving Global Outcomes acutekidney injury work group (2012). KDIGO clinical practice guideline for acute kidney injury. Kidney Int Suppl.

[15] Tsujimoto H (2020). Pharmacological interventions for preventing clotting of extracorporeal circuits during continuous renal replacement therapy. Cochrane Database Syst Rev.

[16] Zarbock A (2020). Effect of regional citrate anticoagulation vs systemic heparin anticoagulation during continuous kidney replacement therapy on dialysis filter life span and mortality among critically ill patients with acute kidney injury: a randomized clinical trial. JAMA.

[17] Tsukagoshi S (2001). [Molecular specificity of nafamostat mesilate (FUT), a drug used for the treatments of DIC and acute pancreatitis and as an anticoagulant–the pharmacodynamics and pharmacological action]. Gan To Kagaku Ryoho.

[18] Chen PY, Tsiatis AA (2001). Causal inference on the difference of the restricted mean lifetime between two groups. Biometrics.

[19] Royston P, Parmar MKB (2011). The use of restricted mean survival time to estimate the treatment effect in randomized clinical trials when the proportional hazards assumption is in doubt. Stat Med.

[20] Sato T, Matsuyama Y (2003). Marginal structural models as a tool for standardization. Epidemiology.

[21] Rosenbaum PR, Rubin DB (1983). The central role of the propensity score in observational studies for causal effects. Biometrika.

[22] Baek NN (2012). The role of nafamostat mesylate in continuous renal replacement therapy among patients at high risk of bleeding. Ren Fail.

[23] Lee YK (2014). Ability of nafamostat mesilate to prolong filter patency during continuous renal replacement therapy in patients at high risk of bleeding: a randomized controlled study. PLoS ONE.

[24] Miyaji MJ (2022). Comparison of nafamostat mesilate to citrate anticoagulation in pediatric continuous kidney replacement therapy. Pediatr Nephrol..

[25] Lim JY (2016). Anticoagulation during extracorporeal membrane oxygenation; nafamostat mesilate versus heparin. Ann Thorac Surg.

[26] Park JH (2015). Nafamostat mesilate as a regional anticoagulant in patients with bleeding complications during extracorporeal membrane oxygenation. Int J Artif Organs.

[27] Makino S (2016). Comparison of nafamostat mesilate and unfractionated heparin as anticoagulants during continuous renal replacement therapy. Int J Artif Organs.

[28] Hwang SD (2013). Nafamostat mesilate for anticoagulation in continuous renal replacement therapy. Int J Artif Organs.

[29] Yang JW (2009). Superior outcome of nafamostat mesilate as an anticoagulant in patients undergoing maintenance hemodialysis with intracerebral hemorrhage. Ren Fail.

[30] Zhang W (2021). Continuous renal replacement therapy without anticoagulation in critically ill patients at high risk of bleeding: a systematic review and meta-analysis. Semin Dial.

[31] Ohtake Y (1991). Nafamostat mesylate as anticoagulant in continuous hemofiltration and continuous hemodiafiltration. Contrib Nephrol.

