# Correction: Unfractionated heparin versus nafamostat mesylate for anticoagulation during continuous kidney replacement therapy: an observational study

**DOI:** 10.1186/s12882-023-03067-8

**Published:** 2023-01-26

**Authors:** Shinya Kameda, Tomoko Fujii, Junpei Ikeda, Akira Kageyama, Toshishige Takagi, Naoki Miyayama, Kengo Asano, Arata Endo, Shoichi Uezono

**Affiliations:** 1 Intensive Care Unit, The Jikei University Hospital, 3-19-18 Nishi-Shimbashi, Minato-Ku, Tokyo, 105-8471 Japan; 2 Department of Clinical Engineering Technology, The Jikei University Hospital, Tokyo, Japan; 3 Department of Pharmacy, The Jikei University Hospital, Tokyo, Japan; 4 Department of Anesthesiology, The Jikei University Hospital, Tokyo, Japan

**Correction: BMC Nephrol (2023) 24:12**

**https://doi.org/10.1186/s12882-023-03060-1**

Following publication of the original article [1], we have been informed that Figs. 1 and 2 were switched. Figure 1 should contain 8 components (a. pH to h. chloride) and Fig. 2 should be the curve.

The original article has been corrected.
