# Supplementary material for: Unfractionated heparin versus nafamostat mesylate for anticoagulation during continuous kidney replacement therapy: an observational study
Source: BMC Nephrol. 2023 Jan 16;24:12. doi: 10.1186/s12882-023-03060-1 (PMC9840945; doi:10.1186/s12882-023-03060-1)
Supplement: Supplementary file 1 — Additional file 1: eFigure 1. Standardized mean differences of variables before and after propensity score matching. eFigure 2. Distribution of propensity scores. eTable 1. Dialysis settings and vital signs at the start of CKRT. eTable 2. Arterial blood gas analysis at the start of CKRT, 5 hours, and 24 hours (mean SD). eTable 3. Sensitivity analysis with SMRW data (reference group = nafamostat mesylate). eTable 4. Post-hoc sensitivity analyses and power calculations. [file 12882_2023_3060_MOESM1_ESM.pdf]

eFigure 1. Standardized mean differences of variables before and after propensity score matching.

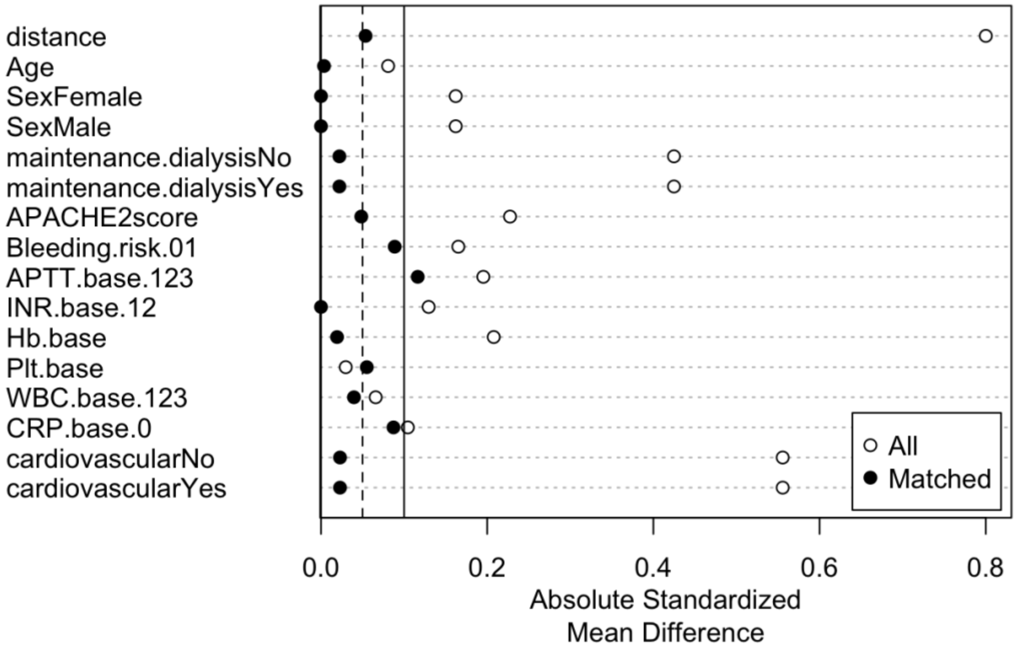

eFigure 2. Distribution of propensity scores.

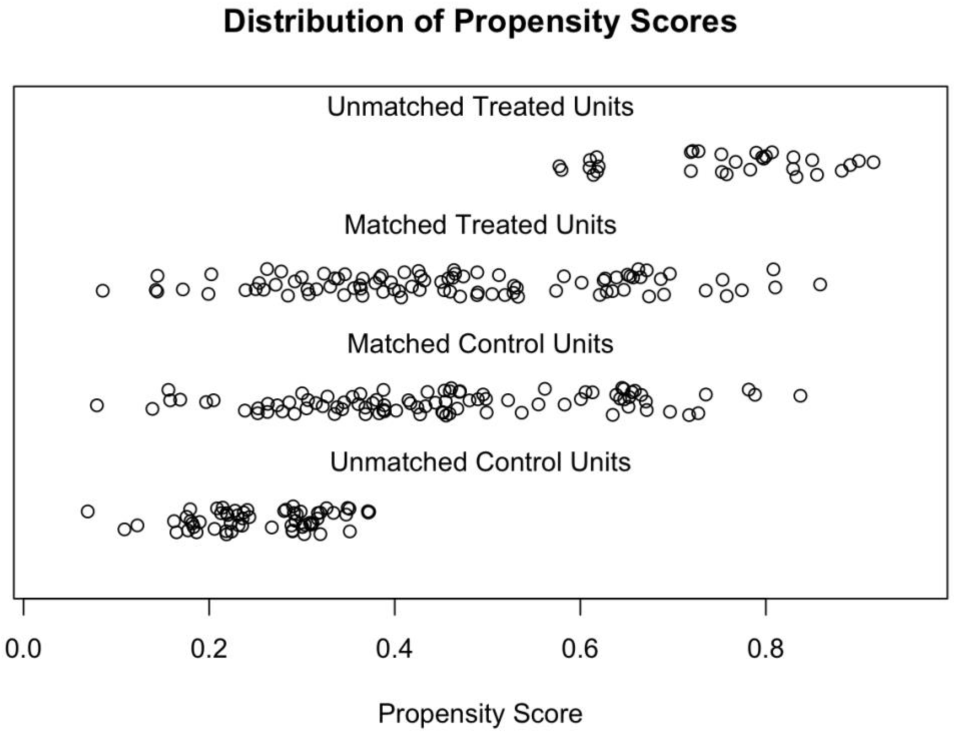

eTable 1. Dialysis settings and vital signs at the start of CKRT.

	Nafamostat mesylate (n=129)	Unfractionated heparin (n=157)	p-value
Quantity of blood flow: QB, ml/min	99 (6)	99 (9)	0.936
Quantity of dialysate flow rate: QD mL/kg/min	17.5 (12.5)	14.2 (8.0)	0.019
Fluid removal rate: UFR, mL/h	38.2 (89.2)	32.1 (109.7)	0.664
Mean arterial pressure, mmHg	80 (18)	77 (19)	0.124
Heart rate, bpm	96 (21)	91 (18)	0.025
Respiratory rate, /min	21 (7)	20 (7)	0.142

Means and the standard deviations are reported.

eTable 2. Arterial blood gas analysis at the start of CKRT, 5 hours, and 24 hours (mean SD).

	Nafamostat mesylate（n=129）			Unfractionated heparin(n=157)		
	0 h	5 h	24 h	0 h	5 h	24 h
FIO_2	42.8 (21.8)	42.5 (20.7)	36.7 (15.6)	43.6 (23.3)	38.3 (16.9)	35.1 (16.7)
pH	7.33 (0.12)	7.34 (0.10)	7.38 (0.08)	7.34 (0.09)	7.36 (0.07)	7.39 (0.06)
CO_2 (mmHg)	35.5 (10.7)	35.9 (10.5)	36.2 (10.3)	37.4 (8.7)	37.2 (8.0)	36.7 (6.5)
O_2 (mmHg)	112. 5(53.0)	105.3 (38.1)	95.3 (21.3)	114.3 (58.5)	103.5 (31.5)	98.2 (29.4)
HCO_3 (mmol/L)	18.4 (5.7)	19.0 (5.0)	21.0 (4.1)	19.6 (4.4)	20.5 (3.6)	21.7 (2.6)
BE (mmol/L)	-6.6 (6.7)	-5.7 (5.6)	-3.2 (4.4)	-5.3 (5.1)	-4.0 (3.8)	-2.4 (2.8)
Na (mmol/L)	136.8 (7.0)	136.8 (6.2)	136.8 (4.7)	135.5 (5.0)	135.5 (4.8)	135.8 (3.8)
K (mmol/L)	4.8 (1.0)	4.6 (0.8)	4.3 (0.7)	4.9 (0.9)	4.7 (0.8)	4.3 (0.6)
CL (mmol/L)	106.6 (7.6)	106.9 (6.5)	106.1 (4.4)	105.7 (5.8)	106.4 (5.3)	105.3 (3.9)
iCa (mmol/L)	1.11 (0.12)	1.13 (0.11)	1.16 (0.10)	1.13 (0.12)	1.14 (0.11)	1.18 (0.10)
Lac (mmol/L)	3.0 (3.7)	2.6 (3.1)	2.4 (2.8)	2.8 (3.6)	2.1 (2.5)	1.6 (1.4)

eTable 3. Sensitivity analysis with SMRW data (reference group = nafamostat mesylate)

	Mean difference or Risk difference	95% CI		p-value
Filter life, day	0.52	0.09	0.96	0.017
ICU length of stay, day	-0.78	-4.65	3.08	0.691
Hospital length of stay, day	2.5	-17.1	22.2	0.801
ICU death, %	-6.9	-19.8	6.1	0.300
Hospital death, %	-4.0	-18.4	10.4	0.589
Mechanical ventilation days, day	-0.3	-3.6	3.0	0.851
Red blood cell over 48 hours, ml	-12.23	-83.61	59.16	0.737
Platelet over 48 hours, ml	-6.05	-29.07	16.97	0.606
Fresh frozen plasma over 48 hours, ml	-55.05	-125.48	15.38	0.125
C-reactive protein at 24 hours, mg/dL	0.20	-2.32	2.73	0.871
Dialysis dependence at hospital discharge among survivors, %	0.18	0.06	0.31	0.004
Creatinine at hospital discharge among non-dialysis dependent patients, mg/dL	-0.18	-0.92	0.56	0.633

eTable 4. Post-hoc sensitivity analyses and power calculations

Filter life in the matched cohort was longer in the UFH group than in the NM group when inversed probability of censoring weight was applied.<br>Mean difference, 0.59 days, 95% CI: 0.20–0.98, p = 0.002.	
Power analysis of the study sample size (alpha = 0.05, d = 0.5, two sided)	
Overall cohort (n1 = 129, n2 = 157)	98.7%
Matched cohort (n1 = n2 = 91)	91.8%
